# Dilemmas in the Clinical Management of pT1 Colorectal Cancer

**DOI:** 10.3390/cancers15133511

**Published:** 2023-07-06

**Authors:** Diana Zaffalon, Maria Daca-Alvarez, Karmele Saez de Gordoa, María Pellisé

**Affiliations:** 1 Gastroenterology Department, Institut d’Investigacions Biomèdiques August Pi i Sunyer (IDIBAPS), Centro de Investigación Biomédica en Red de Enfermedades Hepáticas y Digestivas (CIBEREHD), Hospital Clínic de Barcelona, Villarroel 170, 08036 Barcelona, Spain; dzaffalon@cst.cat (D.Z.); daca@clinic.cat (M.D.-A.); 2 Gastroenterology Department, Consorci Sanitari de Terrassa, Torrebonica, s/n, 08227 Terrassa, Spain; 3 Pathology Department, Centre de Diagnostic Biomèdic, Hospital Clínic de Barcelona, Villarroel 170, 08036 Barcelona, Spain; saezdegord@clinic.cat

**Keywords:** pT1 CRC, colorectal cancer, polyp, colonoscopy, polypectomy, TAMIS, minimally invasive surgery, prognosis, adverse events, histological risk factors, disease-free survival, colorectal surgery

## Abstract

**Simple Summary:**

Population-based colorectal cancer screening programs have increased the incidence of pT1 colorectal cancer. These incipient invasive cancers have a very good prognosis and can be treated locally, but more than half of these cases are treated with surgery due to the presence of histological criteria associated with the presence of lymph node metastasis. Only 2–10.5% of those have lymph node metastasis, and the residual tumor is present in less than 20%, leading to overtreatment. Additional surgery increases the risk of morbidity and mortality, and recent evidence shows that it may not impact the prognosis for pT1. This article reviews the limitations of histological evaluation, treatment modalities and prognosis, adverse effects, and new possibilities of endoscopic treatment.

**Abstract:**

Implementation of population-based colorectal cancer screening programs has led to increases in the incidence of pT1 colorectal cancer. These incipient invasive cancers have a very good prognosis and can be treated locally, but more than half of these cases are treated with surgery due to the presence of histological high-risk criteria. These high-risk criteria are suboptimal, with no consensus among clinical guidelines, heterogeneity in definitions and assessment, and poor concordance in evaluation, and recent evidence suggests that some of these criteria considered high risk might not necessarily affect individual prognosis. Current criteria classify most patients as high risk with an indication for additional surgery, but only 2–10.5% have lymph node metastasis, and the residual tumor is present in less than 20%, leading to overtreatment. Patients with pT1 colorectal cancer have excellent disease-free survival, and recent evidence indicates that the type of treatment, whether endoscopic or surgical, does not significantly impact prognosis. As a result, the protective role of surgery is questionable. Moreover, surgery is a more aggressive treatment option, with the potential for higher morbidity and mortality rates. This article presents a comprehensive review of recent evidence on the clinical management of pT1 colorectal cancer. The review analyzes the limitations of histological evaluation, the prognostic implications of histological risk status and the treatment performed, the adverse effects associated with both endoscopic and surgical treatments, and new advances in endoscopic treatment.

## 1. Introduction

Colorectal cancer (CRC) is the third most common cancer in Western countries and the second most frequent cause of cancer death for both sexes in Spain [1]. Endoscopic resection of CRC precursor lesions (polypectomy) decreases CRC incidence and mortality [2]. pT1 CRC is a tumor that generally grows inside a polyp and invades the muscularis mucosae but not beyond the submucosal layer. It represents the earliest stage of CRC. The incidence of T1 CRC is increasing due to screening programs, and 40% of all screen-detected cancers are stage T1 [3,4].

Polyps with intramucosal carcinoma do not metastasize because there are no lymphatic vessels above the muscularis mucosa. They are thus curable with endoscopic polypectomy. When the malignant cells penetrate the muscularis mucosa, the risk of lymphatic invasion and, therefore, of metastasis is believed to progressively increase as the tumor penetrates deeper into the submucosal layer. The optimal treatment for this type of lesion is controversial. Characteristically, the submucosa of the colon has few lymphatic vessels on the surface, which means that some incipient invasive cancers have a very good prognosis and can be treated locally. Thus, endoscopic resection of pT1 CRCs is an attractive option because it is more conservative in terms of organ preservation and is associated with lower morbidity, mortality, and costs than surgery. However, from an oncological point of view, this approach does not include the resection of the locoregional lymph nodes, and this would be a suboptimal treatment in patients with a significant probability of lymph node metastasis (LNM). Therefore, more than half of all cases of this early-stage cancer, potentially curable with endoscopic treatment, are treated with surgery in clinical practice, even though this is the treatment applied to the most advanced stages of CRC [3].

The ultimate decision on whether to proceed with additional surgery after primary endoscopic treatment for pT1 CRC is primarily based on histology. Current histological risk criteria for pT1 CRC are suboptimal, with no consensus among clinical guidelines. Moreover, recent evidence suggests that some of these criteria deemed high risk may not necessarily affect prognosis on their own. To our knowledge, no clinical trials have compared a wait-and-see strategy and secondary surgery after endoscopic resection. It must be taken into account that screening programs include healthy individuals and that the disease-free survival of patients with early-stage CRC is excellent, above 90% [5,6,7]. Using the current criteria, most patients are classified as high-risk, which leads to additional surgical treatment. This may reduce locoregional recurrence, lymphatic spread, and cancer-related death but also entails an increased risk of morbidity, mortality, and functional loss and added treatment-related costs that should be considered in the clinical scenario of early-stage cancer with a good prognosis.

In this review article, we analyze three of the many dilemmas regarding the clinical management of pT1 CRC.

## 2. Suboptimal Histological Criteria Lead to Overtreatment

The final treatment decision for pT1 CRC, namely, if additional surgery is required or not after primary endoscopic treatment, is primarily based on histology. The so-called histological high-risk factors are believed to confer an increased risk of LNM that ranges from 0.7% to 36.4% [8]. The factors associated with the presence of LNM vary among clinical guidelines. Currently, pT1 CRC with any of the following histological findings is considered high risk for LNM and is indicated for additional surgery: lymphovascular invasion (LVI), poorly differentiated histology (PD), presence of tumor budding (TB), positive vertical margin, or deep submucosal invasion (DSI).

There is consensus in the guidelines for three factors: PD, TB, and LVI. The other risk factors, DSI and positive vertical margin, are described in the guidelines, but recent evidence suggests that they should not be considered independent high-risk factors. The histological risk criteria according to the main clinical practice guidelines are summarized in Table 1 [9,10,11,12,13].

### 2.1. Risk Factors with Consensus

○ Lymphatic and/or vascular invasion: LVI is the risk factor most strongly correlated with LNM and poor outcome [14,15,16] but is also known for high interobserver variability [17]. LVI can be assessed using hematoxylin and eosin staining with or without immunohistochemistry. A meta-analysis reported a clear benefit of using immunohistochemistry for predicting LMN, with an increase in the detection of LVI from 14.3% to 35.7%, but it is used only in doubtful cases in clinical practice [18];
○ Poorly differentiated histology: Similar to LVI, PD is an established risk factor, with recent evidence suggesting that it is an independent risk factor associated with poor prognosis [16]. As with LVI, PD has high interobserver variability, with the lowest value of kappa (0.07) in a concordance study of histological assessment in pT1 CRC [19]. Due to highly variable reporting of the tumor grade according to the three-tiered system (G1–G3), the latest version of the WHO classification [20] recommends a two-tiered system, where G1 and G2 are combined as low grade and G3 is considered high grade. This change is based on the similar prognosis for G1 and G2 tumors and improved reproducibility. However, variability remains in how this new system is applied in clinical practice [21,22];
○ Tumor budding: Defined as a single tumor cell or cell cluster of four tumor cells or less extending at the invasive margin of the cancer. TB is an established predictor of LNM in pT1 CRC according to the guidelines [23]. Nevertheless, it is reported in less than 50% of published studies [22]. Also, TB and tumor grade are different concepts, and there is no consensus in the guidelines on the grade of TB that confers a worse prognosis.

### 2.2. Risk Factors without Consensus

○ Deep submucosal invasion: Recent evidence calls into question whether DSI confers a risk, given that it seems to not be an independent risk factor for LNM. A recent meta-analysis published by a Dutch T1 CRC group [24] that included 67 studies (21,238 patients) showed that DSI, as a solitary risk factor, only has an absolute risk of LNM of 2.6% and was not a significant predictor of LNM in a multivariable meta-analysis (odds ratio [OR], 1.73; 95% confidence interval [CI], 0.96–3.12), in contrast to a significant association of LNM with PD (OR, 2.14; 95% CI, 1.39–3.28), high-grade TB (OR, 2.83; 95% CI, 2.06–3.88), and LVI (OR, 3.16; 95% CI, 1.88–5.33) (24). The authors concluded that DSI should be reconsidered as a strong indicator for oncological surgery;
○ Resection margin: A positive margin (R1) is considered a risk factor in most guidelines, although the definition of this item is controversial. The different definitions of an R1 margin include cancer that is within the diathermy margin, ≤0.1 mm from the margin, ≤1 mm from the margin, and ≤2 mm from the margin [22]. In addition, the definition of a positive margin may depend on the morphology (sessile or pedunculated polyp) [11,12,13]. The most common definition for R1 is <1 mm, based on studies showing a risk of residual disease of 6.1–21% [25], but recent evidence questions this suggestion, as it shows similar risk for residual disease in patients with resection margins between 0.1 mm (2.9%; 95% CI, 1.0–6.7%) and 1 mm (0.6%; 95% CI, 0.1–2.1%), in the absence of other histological risk factors [26].

Regarding the endoscopic resection technique, the risk of residual disease may be more strongly related to incomplete resection than margin status. Table 2 shows the risk of residual disease in patients with pT1 CRC treated by endoscopy with subsequent rescue treatment (surgery or local). Studies that report residual disease above 15% often have high rates of incomplete endoscopic resections (over 10%). Conversely, studies with lower residual disease values (0.6–8.2%) mostly exclude cases of incomplete resection. Additionally, the evaluation of the resection margin is influenced by the technique. Conventional polypectomy is usually confined to the superficial or middle submucosal layer and is usually fragmented when the polyp is greater than 20 mm. An indeterminate margin (Rx) is usually reported in fragmented resections, even if it is a complete resection. An Rx margin can be mistaken for an incomplete resection, with an indication for salvage surgery. A more conservative strategy would be close endoscopic surveillance, instead of additional surgery, in cases of a doubtful margin, but only if the endoscopic resection is considered complete and there are no other high-risk factors.

Suboptimal histological criteria have repercussions in clinical practice. By using the current clinicopathological criteria, 60–70% of pT1 CRC patients are classified as high risk, but the post-surgical pathological results show that only 2–10.5% have LNM and that residual tumor is present in less than 20% [6,27,28,29,30,31,32], which leads to overtreatment in more than 80% of cases. Associations between histological risk factors and the presence of LNM have been reported in several meta-analyses, with the limitation that they were based on retrospective studies with different definitions of histological risk factors [15,33]. Additionally, these factors can appear simultaneously, and it is, therefore, difficult to know the real weight of each individual one.

Current histopathological criteria have important limitations regarding their definitions, assessment, poor concordance, and lack of reproducibility. Davenport et al. reported a significant variation in the assessment of important prognostic parameters in pT1 CRC by four expert gastroenterology pathologists in the UK CRC screening program, with kappas ranging from 0.07 for tumoral differentiation to 0.15 for the Haggitt level, and 0.35 for LVI (poor to fair agreement) [19]. In the Dutch CRC screening cohort, a panel of experts reviewed pT1 CRC patients; discrepancies were identified in 53.0% of cases and could have led to alternative treatment approaches in 30.1% [17].

**Table 2:** Risk of residual disease in patients with pT1 CRC treated with secondary surgery after endoscopic treatment. Retrospective study series.

Author	Year	N	Histological Risk Criteria	Residual Disease	Endoscopic Resection	Resection Margin
Overwater Dutch cohort [6]	2018	339	High-risk histology	52/339 (15.3%)	Incomplete ER 39/339 (11.5%)	NA
Richards Scottish BSP cohort [31]	2018	186	NA ^1^	41/186 (22%)	Incomplete ER 189/485 (39%)	NA
Eun Hye Oh Korean cohort [32]	2021	464	High-risk histology	29/464 (6.8%)	Incomplete ER 40/464 (8.6%)	261/464 (56.3%) Rx/R1 29 cases of residual disease: Rx/R1
Levic Danish CRC group [28]	2019	268	NA (low and high risk, unknown proportion)	22/268 (8.2%)	Incomplete ER excluded	22/218 (10%) Rx/R1
Yamaoka Japanese cohort [27]	2020	244	High-risk histology	11/244 (4.5%)	Incomplete ER excluded	54/244 (22.1%) Rx/R1
Backes Dutch cohort [30]	2017	358	Low risk: 19 (13.6%) High risk: 287 (58.8%) Missing: 57	11/358 (3.1%)	Incomplete ER excluded	241/358 (67.3%) Rx/R1
Gijsbers Dutch cohort ^2^ [26]	2022	171	Low risk: No LVI No PD	5/171 (2.9%)	Incomplete ER excluded	Free margin 0.1–1 mm
		351		2/351 (0.6%)		Free margin > 1 mm

ER, endoscopic resection; NA, not assessed. ^1^. In these retrospective series based on the screening program, it can be hypothesized that patients undergoing secondary surgery meet high-risk histological criteria, but it is not specified. ^2^. In this cohort, the residual disease is defined as the presence of tumor tissue in the rescue treatment specimen and evidence of local recurrence during follow-up (median, 10 months).

The integration of digital pathology into clinical practice represents an opportunity to enhance histological risk assessment. By incorporating artificial intelligence (AI) for automated analysis, pathologist subjectivity could be reduced, leading to more objective results. Predictive models could also be a useful resource for individualized risk estimation. For example, Kudo et al. [34] developed a predictive model using machine learning that incorporates clinical, endoscopic, and histological variables. The model was found to more accurately predict the presence of LNM compared to traditional clinical guidelines. Another group from the US developed a predictive model that uses a microRNA signature in conjunction with histological criteria. This model enabled the reclassification of a high-risk cohort identified by conventional histological criteria, resulting in a reduction of truly high-risk patients from 100% to only 25%. This approach could have prevented overtreatment (additional surgery) in 92% of cases, reducing the need for only 18% of cases [35]. Both of these predictive models need to be validated in prospective cohorts.

## 3. CRC pT1 Survival Appears to Depend on Histology, Not Treatment

Traditionally, it has been believed that surgery prevents recurrence and reduces mortality associated with CRC but, taking into consideration the fact that the five-year overall disease-free survival rate for CRC T1 CRC is 94% [29], the potential benefit of surgery remains marginal and the protective role of surgery is questionable. The relevant clinical guidelines were based on retrospective studies focused on risk factors for LNM as the main outcome. Two recent meta-analyses focused on the most important outcomes: recurrence and survival (Table 3).

The meta-analysis by Antonelli et al. [36] obtained pooled incidences of recurrence and CRC-specific mortality of 9.5% (6.7–13.3%) and 3.8% (2.4–5.8%), respectively, for high-risk lesions and of 1.2% (0.6–2.5%) and 0.6% (0.2–1.7%) for low-risk lesions. The more recent meta-analysis by Dang et al. [37] that included 71 studies and 5167 endoscopically treated patients with pT1 CRC showed similar results: risk of recurrence after endoscopic treatment of 3.3%, with pooled incidences of CRC recurrence of 7.0% for high-risk T1 CRCs (4.9–9.9%) and 0.7% (0.4–1.2%) for low-risk T1 CRCs. Regarding CRC-specific mortality, the pooled incidence was 4.5% (95% CI, 3.2–6.3%) for high-risk status patients, with 40% of patients with recurrence dying from the disease. The results of these meta-analyses show a worse prognosis for histological risk status, despite the above-mentioned limitations of these criteria.

According to recent evidence, it is becoming clear that endoscopic treatment before surgery does not affect outcomes in high-risk patients regarding survival, recurrence, and postoperative complications [38,39], but it is unclear whether surgery reduces recurrence and mortality from pT1 CRC. Several studies suggest that recurrence and disease-free survival do not change with the treatment modality performed. In a recent meta-analysis by Yeh et al. [29], based on 17 retrospective studies with 19,979 patients, a median follow-up of 36 months, and the inclusion of cases with high-risk histological status, no significant differences were found between endoscopic resection and primary surgery in overall survival (79.6% vs. 82.1%; hazard ratio [HR], 1.10; 95% CI, 0.84–1.45), recurrence-free survival (96.0% vs. 96.7%; HR, 1.28; 95% CI, 0.87–1.88), or disease-specific survival (94.8% vs. 96.5%; HR, 1.09; 95% CI, 0.67–1.78).

Remarkably, patients with high-risk histology, who typically receive primary or secondary surgery due to their heightened risk of recurrence and mortality, do not appear to gain any significant advantages from this supplementary treatment. One potential reason for this could be that the likelihood of an unfavorable prognosis is more related to the aggressiveness of the tumor than to the treatment received. It is important to note that pT1 CRC has an excellent prognosis, and the infrequency of events, such as recurrence and mortality, can make it challenging to establish the efficacy of surgery in improving patient outcomes. Additionally, while surgery can be highly beneficial for certain selected patients, this subset of individuals is not as prevalent, resulting in a lower overall impact that may not be apparent in published studies. Long-term prospective follow-up studies with large sample sizes are needed to resolve this issue since recurrence and mortality secondary to pT1 CRC are very rare events.

## 4. Overtreatment Leads to a Higher Chance of Adverse Events

More than half of patients with CRC pT1 undergo surgical treatment. The estimated number of pT1 CRC treated with surgery, according to population screening programs in Europe, is 38–55% [5,7,31]. Also, the rates of surgery for non-malignant colorectal polyps have shown a significant increase over time, rising from 5.9 per 100,000 adults in 2000 to 9.4 per 100,000 adults in 2014, according to Peery et al. [40]. This trend can be extended in the case of malignant polyps.

In this clinical setting, healthy participants are diagnosed with a malignant polyp, which is potentially curable with local treatment. Nevertheless, the patient is more likely to end up in surgery, with all of the associated costs and risk of adverse events (AEs), a disheartening outcome in a previously healthy asymptomatic patient. Evidence shows that surgery has a higher risk of morbidity and mortality than endoscopy treatment [29]. It is difficult to compare AEs between the two treatment modalities because the only studies to report complications after endoscopic resection of pT1 CRC were retrospective, and there is wide variability in definitions. Perforation, bleeding, and post-polypectomy syndrome (PPES) are the most commonly reported AEs, and nearly no studies report complications related to sedation. Complications after the surgical treatment of pT1 CRC have been better studied.

In the Yeh et al. meta-analysis [29], a significantly lower proportion of patients who underwent primary endoscopic resection had procedure-related AEs (2.3%) compared with patients who underwent primary surgery (10.9%) (*p* < 0.01). AEs after the endoscopic treatment of pT1 CRC are shown in Table 4.

Regarding AEs after endoscopic treatment, the retrospective Van de Ven et al. study [41] specifically assessed this outcome. A 5.5% rate of AEs was reported. The most common AEs were postprocedural bleeding (3.7%), followed by perforation (1.2%) and PPES (0.6%). Compared to the incidence rates of AEs after the endoscopic resection of larger adenomas, the rates of postprocedural bleeding (4.7–10.9%) and perforation (1.2%) are similar [45]. It seems that there is no additional risk in the endoscopic resection of pT1 CRC compared with conventional adenomas.

AEs after the surgical treatment of pT1 CRC are summarized in Table 5. The rate of AEs (morbidity) after surgical treatment is around 20%, with rates of serious AEs (Clavien–Dindo ≥ III) of 8% and anastomotic leak of 4% [46]. Regarding mortality, Belderbos et al. [7] reported a 30-day mortality rate of 3.2% in patients undergoing initial surgical resection, which was significantly higher than that of endoscopic resection (1.4%) (*p* = 0.016). Indeed, the mortality rate after endoscopic treatment is reported to be zero in most published studies. Comparing surgery for pT1 cancer with that performed for more advanced CRC stages (T2–T3), the rates of complications, severe complications, and mortality are similar [46]. 

Multidisciplinary decision-making and individualized perioperative risk assessment are crucial in deciding which patients will benefit from surgical treatment. Multiple scales have been described that can help assess comorbidities and life expectancy prior to deciding on optimal treatment. One of the most used is the Charlson Comorbidity Index. A study evaluating the results after endoscopic mucosal resection in elderly patients shows an age of more than 79 years and a Charlson index >3 were associated with shortened survival [47]. The modified frailty index is a simple score that can be useful in evaluating the degree of frailty and predicting the risk of postoperative AEs. This score has been evaluated in colorectal surgery, and frail patients (score of ≥2) had more risk of developing 30-day AEs: overall morbidity, mortality, prolonged length of hospital stay, non-home discharge, reoperation, and readmission but, more importantly had lower disease-free survival and overall survival rates [48]. Vermeer et al. [46] specifically evaluated factors associated with post-surgical AEs in pT1 CRC. Male sex, cardiac comorbidity, ASA grades III–IV, previous abdominal surgery, open approach, and subtotal colectomy were associated with an increased risk of severe complications. These findings were used to establish a risk stratification, and men with ASA grade III-IV undergoing right or left colectomy faced the highest risk of severe complications. When considering primary or additional oncological surgery for a patient with rectal pT1 that is accessible through local techniques, caution should be exercised, particularly in advanced-age men with high comorbidities, due to the high complication rates [46]. In the context of pT1 CRC, which is early-stage cancer with a favorable prognosis, it is important to consider local conservative treatments for frail patients who are at risk of experiencing post-surgical AEs. This consideration could be extended for fragile histological high-risk patients as well since the risk of complications may outweigh the risk of cancer mortality.

There are several methods to treat CRC locally and minimally invasively: polypectomy, endoscopic submucosal dissection (ESD) [44,49], endoscopic intermuscular dissection (EID) [50], endoscopic full-thickness resection (eFTR) [51,52], transanal microsurgery (TAMIS), and mixed local resections by laparoscopic and endoscopic procedures. Local excision using transanal microsurgery (TAMIS) carries a lower risk of morbidity, at just 11% [53,54], versus the 22% rate of severe complications [46] described for rectal surgery. If we compare endoscopic en bloc resection and local excision for pT1 CRC, there is still insufficient evidence to favor one option over the other. The two published meta-analyses [55,56] comparing ESD and transanal microsurgery found no differences between R0 or en bloc resection and the rate of AEs. The results of a multicenter randomized controlled trial (TRIASSIC) will provide more evidence in this regard [57].

Regarding developments in endoscopic treatments for pT1 CRC, the two emerging techniques are:

○ Endoscopic intermuscular dissection (EID): Involves dissection between the circular and longitudinal layers of the muscolaris propria for rectal tumors. In a prospective cohort study of 67 lesions, en bloc resection was possible in 96% of cases (95% CI, 89–99%), with R0 in 81% (95% CI, 70–89%). Eight AEs were reported (12%): six minor AEs treated conservatively, one case of delayed bleeding, and one of rectal stenosis that required dilatation [50];
○ Endoscopic full-thickness resection (eFTR): With an over-the-scope device, this technique has emerged as a local treatment option for pT1 CRCs, with the possibility of en bloc transmural resection that allows for more accurate histological assessment [51,52]. It is currently used in daily practice as the primary treatment for pT1 tumors < 3 cm, with a technical success of 89%. It is also used as a rescue treatment for previous incomplete R1/Rx-resected pT1 and is technically feasible in 85%, with promising short-term results. Regarding AEs, eFTR has a similar rate of AEs and bleeding and a slightly higher perforation rate than conventional endoscopic resection techniques (Table 4) [52].

To our knowledge, no comparative studies have examined these new techniques and conventional endoscopic techniques and surgery in terms of safety and effectiveness. Long-term oncologic safety has not been established.

## 5. Conclusions and Future Directions

Future research efforts should focus on improving the quality of the histological evaluation of pT1 CRC, given that it is such an important part of the decision-making process regarding additional treatments. Histological risk factors could be redefined, based on recent evidence, particularly regarding submucosal invasion not being an independent risk factor and the consideration of a margin >0.1 as a free (R0) margin. Moreover, it is essential to improve concordance in histological evaluations, and AI may play a pivotal role in achieving this goal.

Due to the uncertainty regarding the protective benefits of surgery for high-risk patients, it is crucial to thoroughly assess the need for this treatment as a primary or additional measure on a case-by-case basis. In particular, it may be worth considering closer monitoring after endoscopic treatment in high-risk patients rather than opting for additional surgery in elderly patients with considerable comorbidities or for pT1 CRC located in the rectum, where local excision techniques may be a safer alternative with a lower risk of AEs. Advanced local endoscopic techniques are now available that represent organ-preserving procedures and permit complete resection while allowing better histological assessment. These techniques may offer a greater chance of achieving a curative R0 resection and reducing the risk of AEs in the future.

To advance the management of pT1 CRC, it is imperative to conduct long-term prospective follow-up studies with large sample sizes to resolve the existing dilemmas, especially randomized controlled trials that can compare the treatment strategies, such as surgery versus endoscopic treatment in high-risk patients, as well as evaluate the safety and oncological outcomes of the new endoscopic treatments.

We must acknowledge the limitations of histology and take a comprehensive approach by additionally considering demographic, clinical, endoscopic, and molecular factors when estimating the risk of pT1 CRC. To improve the management of the many clinical dilemmas of pT1 CRC, a multidisciplinary approach should be adopted for decision making. The incorporation of predictive models could further optimize the decision-making process in clinical settings.

## Figures and Tables

**Table 1:** Risk factors for lymph node metastasis according to international guidelines.

	Lymphovascular Invasion	Degree of Differentiation	Submucosal Invasion	Resection Margin	Tumor Budding
JSCCR 2019 [9]	Yes	Poorly differentiated, signet ring or mucinous adenocarcinoma	>1000 μm (T1b)	Yes: Positive vertical margin ^1^	Budding grade 2/3
NCCN 2021 [12]	Yes	Poorly differentiated	Not described	Yes: Positive type unspecified	Yes, suggested Unspecified grade
ESMO 2020 [10]	Yes	Poorly differentiated	Haggit 4 (pedunculated) No clear recommendation for sessile and flat lesions	No risk ^2^	Budding grade 2/3
ESGE-ESDO 2019 [11]	Yes	Poorly differentiated	≥1000 μm Haggit 4 in pedunculated SM2–3 in non-pedunculated	Yes: Positive margin (<1 mm) or cannot be assesed	Intense tumor budding Unspecified grade
ASGE 2020 [13]	Yes	Poorly differentiated	>1000 μm in non-pedunculated No risk in pedunculated	Yes: Positive margin in non-pedunculated <1 mm in pedunculated	Yes: Unspecified grade Only in non-pedunculated

^1^ Positive vertical margin is defined as carcinoma exposed at the submucosal margin of the resected specimen by JSCCR guidelines. ^2^. Positive resection margin (<1 mm) is considered only a risk for local recurrence in ESMO guidelines. Its recommended management comprises additional excision or local surveillance.

**Table 3:** Endoscopic resection without complementary surgery and ≥12 months of follow-up.

Author	High-Risk Status	Low-Risk Status	Total
Antonelli 2019 [36]	5 studies 571 patients	7 studies 650 patients	8 studies
	Recurrence 9.5% (6.7–13.3%) Mortality from CRC 3.8% (2.4–5.4%)	Recurrence 1.2% (0.6–2.5%) Mortality from CRC 0.6% (0.2–1.7%)	Recurrence 4.9% Mortality from CRC 1.5%
Dang 2022 [37]	28 studies 1499 patients	36 studies 1023 patients	71 studies 5167 patients
	Recurrence 7.0% (4.9–9.9%) Mortality from CRC 4.5% (3.2–6.3%)	Recurrence 0.7% (0.4–1.2%) Mortality from CRC 0.1% (0.0–0.7%)	Recurrence 3.3% (2.6–4.3%) Mortality from CRC 1.7% (1.2–2.25%)

**Table 4:** Adverse events (AEs) after the endoscopic treatment of T1 CRC in retrospective series.

Author	Year	N	Type of ER	All AEs	Bleeding	Perforation	PPES	30-Day Mortality
Van de Ven [41]	2020	1069	EMR, ESD, snare resection	59/1069 (5.5%)	40/1069 (3.7%)	13/1069 (1.2%)	6/1069 (0.6%)	0%
Levic [28]	2019	692	Unknown	21/692 (3%)	18/692 (2.5%)	2/692 (0.3%)	NA	NA
Yamaoka [27]	2020	244	EMR, ESD, snare resection	NA	NA	3/244 (1.2%)	NA	NA
Eun Hye Oh [32]	2021	464	EMR, hybrid ESD, ESD	NA	NA	13/464 (2.8%)	NA	NA
Watanabe [42]	2018	110	ESD	7/110 ^1^ (6.3%)	2/110 (1.8%)	5/110 (4.5%)	NA	NA
Grainville [5]	2020	126	Snare resection, EMR	13/126 ^2^ (10.3%)	11/126 (8.7%)	1/126 (0.8%)	NA	NA
Overwater [6]	2018	339	Snare resection, EMR, ESD, TEM	22/339 (6.5%)	14/339 (4.1%)	7/339 (2.1%)	1/339 (0.3%)	1/339 ^3^ (0.3%)
Kim [43]	2015	87	EMR, ESD	5/87 (5.7%)	NA	2/87 (2.3%)	1/87 (1.1%)	NA
Belderbos [7]	2017	370	Snare resection, EMR	NA	NA	NA	NA	5/370 (1.4%)
Zwager [44]	2021	320	eFTR	26/320 (8.1%)	11/320 (3.4%)	11/320 (3.4%)	NA	0%

eFTR, endoscopic full-thickness resection; EMR, endoscopic mucosal resection; ESD, endoscopic submucosal dissection; NA, not assessed; PPES, postpolypectomy electrocoagulation syndrome; TEM, transanal endoscopic microsurgery. ^1^ Only postoperative bleeding and perforation evaluated as adverse events. ^2^ Thirteen patients, with mild, moderate, and severe grades in 8, 3, and 2, respectively, according to ASGE (Cotton reference). ^3^ The only death associated with endoscopy (overwater) was related to a perforation, followed by a cardiovascular complication during emergency surgery.

**Table 5:** Adverse events after the surgical treatment of T1 CRC in retrospective series.

Author	Year	N	Type of Surgery	Mortality	Morbidity	Major Morbidity (Clavien–Dindo ≥ III)	Anastomotic Leak
Richards [31]	2018	186	Colectomy + TEMS (4%)	0%	60/186 (32%)	21/186 (11%)	7/186 (3.8%)
Yamaoka [27]	2020	548	Colectomy	NA	75/548 (13.7%)	24/548 (4.4%)	9/548 (1.6%)
Grainville [5]	2020	163	Colectomy + local excision (7%)	NA	41/163 (25.1%)	12/163 (7.4%)	NA
Overwater [6]	2018	602	Colectomy	15/602 (2.5%)	159/602 (26.4%)	NA	26/602 (4.3%)
Veermer [46]	2019	5170	Colectomy	87/5170 (1.7%)	1219/5170 (23.6%)	427/5170 (8.3%)	176/5170 (3.7%)
Levic [28]	2019	268	Colectomy Subsequent bowel resection	10/268 (3.7%)	55/268 (20.5%)	41/268 (15.3%)	19/268 (7.1%)
Belderbos [7]	2017	725	Colectomy	23/725 (3.2%)	NA	NA	NA

NA, not assessed; TEMS, transanal excision microsurgery.
