# Correction: Zaffalon et al. Dilemmas in the Clinical Management of pT1 Colorectal Cancer. *Cancers* 2023, *15*, 3511

**DOI:** 10.3390/cancers18203327

**Published:** 2026-10-15

**Authors:** Diana Zaffalon, Maria Daca-Alvarez, Karmele Saez de Gordoa, María Pellisé

**Affiliations:** 1Gastroenterology Department, Institut d’Investigacions Biomèdiques August Pi i Sunyer (IDIBAPS), Centro de Investigación Biomédica en Red de Enfermedades Hepáticas y Digestivas (CIBEREHD), https://ror.org/02a2kzf50Hospital Clínic de Barcelona, Villarroel 170, 08036 Barcelona, Spain; 2Gastroenterology Department, https://ror.org/01239b432Consorci Sanitari de Terrassa, Torrebonica, s/n, 08227 Terrassa, Spain; 3Pathology Department, Centre de Diagnostic Biomèdic, https://ror.org/02a2kzf50Hospital Clínic de Barcelona, Villarroel 170, 08036 Barcelona, Spain

## References

With this correction, reference 47 [D’Oria, O.; Golia D’Auge, T.; Baiocco, E.; Vincenzoni, C.; Mancini, E.; Bruno, V.; Chiofalo, B.; Mancari, R.; Vizza, R.; Cutillo, G. The role of preoperative frailty assessment in patients affected by gynecological cancer: A narrative review. *Ital. J. Gynaecol. Obstet.* **2022**, *34*, 76–83.] has been removed from the reference list, and the order of the references has been adjusted accordingly [1]. This removal is due to concerns regarding reviewer conduct. The authors state that the scientific conclusions are unaffected. This correction was approved by the Editor-in-Chief. The original publication has also been updated.
